# Heart failure subphenotypes based on mean arterial pressure trajectory identify patients at increased risk of acute kidney injury

**DOI:** 10.1080/0886022X.2025.2452205

**Published:** 2025-01-19

**Authors:** Xiya Wang, Wenqing Ji, Shuxing Wei, Zhong Dai, Xinzhen Gao, Xue Mei, Shubin Guo

**Affiliations:** a Emergency Medicine Clinical Research Center, Beijing Chao-Yang Hospital, Capital Medical University, Beijing, China; b Beijing Key Laboratory of Cardiopulmonary Cerebral Resuscitation, Beijing, China; c LIANREN Digital Health Co., Ltd, Shanghai, China

**Keywords:** Acute kidney injury, heart failure, mean arterial pressure, group-based trajectory modeling, doubly robust estimation

## Abstract

**Background:**

Acute kidney injury (AKI) is a common complication in heart failure (HF) patients. Patients with heart failure who experience renal injury tend to have a poor prognosis. The objective of this study is to examine the correlation between the occurrence of AKI in heart failure patients and different mean arterial pressure (MAP) trajectories, with the goal of improving early identification and intervention for AKI.

**Methods:**

A retrospective study was conducted on patients with heart failure using data from the Medical Information Mart for Intensive Care IV (MIMIC-IV). We utilized the group-based trajectory modeling (GBTM) method to classify the 24-hour MAP change trajectories in heart failure patients. The occurrence of AKI within the first 7 days of intensive care unit (ICU) admission was considered the outcome. The impact of MAP trajectories on AKI occurrence in heart failure patients was analyzed using Cox proportional hazards models, competing risk models, and doubly robust estimation methods.

**Results:**

A cohort of 8,502 HF patients was analyzed, with their 24-hour MAP trajectories categorized into five groups: Low MAP group (Class 1), Medium MAP group (Class 2), Low-medium MAP group (Class 3), High-to-low MAP group (Class 4), and High MAP group (Class 5). The results from the doubly robust analysis revealed that Class 4 exhibited a significantly increased AKI risk than Class 3 (HR 1.284, 95% CI 1.085–1.521, *p* = 0.003; HR 1.271, 95% CI 1.074–1.505, *p* = 0.005). Conversely, the risks of Class 2 were significantly lower than those of Class 3 (HR 0.846, 95% CI 0.745–0.960, *p* = 0.009; HR 0.879, 95% CI 0.774–0.998, *p* = 0.047).

**Conclusions:**

The 24-hour MAP trajectory in HF patients influences the risk of AKI. A rapid decrease in MAP (Class 4) is associated with a higher AKI risk, while maintaining MAP at a moderate level (Class 2) significantly reduces this risk. Therefore, closely monitoring MAP changes is crucial for preventing AKI in HF.

## Introduction

Heart failure (HF) represents the final phase of cardiovascular disease, with high global morbidity, affecting over 40 million individuals worldwide [1]. Among heart failure patients, acute kidney injury (AKI) is frequently observed as a complication [2]. Data from the Acute Decompensated Heart Failure National Registry (ADHERE) indicate that approximately 30% of hospitalized patients with acute decompensated HF have acute or chronic renal insufficiency [3]. Moreover, heart failure patients with renal injury tend to experience a poor prognosis, with even mild forms of AKI strongly linked to severe clinical outcomes, including a heightened risk of mortality [4–6].

It is challenging for physicians to identify patients who are at risk for AKI. The diagnosis of AKI primarily relies on two key factors: urine output and serum creatinine (Scr) levels. However, Scr was elevated with a delay after kidney injury [7], and urinary output can be influenced by medication effects, making it inadequate for accurately reflecting the severity of renal damage. Previous studies have explored the predictive potential of blood and urinary biomarkers [8] and markers of cardiac injury and congestion [9] in determining the occurrence of AKI. Nevertheless, these studies lacked support from extensive datasets.

Renal hypoperfusion is one of the most common causes of AKI, highlighting the importance of maintaining appropriate blood pressure to prevent AKI. Mean arterial pressure (MAP) serves as a common indicator of optimal blood pressure [10]. Previous research has examined the impact of MAP levels on the incidence of AKI in various clinical scenarios [11–14]. However, these studies did not consider the dynamic nature of MAP.

The group-based trajectory model (GBTM) offers an analytical technique that can summarize MAP records over a period while accounting for the dynamic character of this variable across time [15–17]. It enables the identification of distinct subgroups exhibiting similar longitudinal response patterns [18]. In this research, we classified the 24-h MAP change trajectory in heart failure patients using the GBTM method. Additionally, we investigated the association between different MAP trajectories and the occurrence of AKI in heart failure patients, aiming to enhance early identification and intervention for AKI.

## Methods

### Study design and database

The research was conducted based on a database known as the Medical Information Mart for Intensive Care IV (MIMIC-IV). MIMIC-IV, publicly accessible through PhysioNet, is an extensive open-source medical record database that spans data from 2008 to 2019 [19,20]. The database primarily comprises patient demographics, laboratory test results, vital sign measurements, and comments on prescriptions provided by nurses and doctors. We obtained permission (No. 47937608) to utilize the deidentified data from MIMIC-IV. Due to the anonymized nature of the data, the requirement for informed consent was waived. Our primary purpose is to evaluate the association of MAP subphenotypes with the occurrence of AKI within the initial 7 days of ICU admission.

### Participants and definition

All adult patients diagnosed with HF according to the International Classification of Diseases (ICD) code and admitted to the ICU were eligible for inclusion. We excluded patients (I) who were under the age of 18, (II) complicated by acquired immunodeficiency syndrome, (III) who had a length of stay in the ICU less than 24 h, (IV) who had been diagnosed with end-stage renal disease, (V) with AKI before or within 24 h of admission to the ICU, and (VI) whose missing value of the variables was more than 10%.

The definition of AKI in this study followed the criteria set by the 2012 Kidney Disease Improving Global Outcomes (KDIGO) classification [21]. Given the heterogeneity of urine output measurements and the high degree of missing data, we solely used the Scr component to determine AKI. The lowest creatinine level measured during the 7 days before ICU admission was set as the baseline Scr.

### Variables

Demographic variables, comorbidities, laboratory results, and MAP recordings were collected using Structured Query Language (SQL). Any factors affecting the association between MAP trajectory subtypes and outcomes were considered covariates, selected based on previous studies [22,23] and the biological plausibility of confounders, and they were adjusted for in subsequent analyses. These variables included age, gender, diabetes, hypertension, renal disease, malignant cancer, Charlson Comorbidity Index, body mass index (BMI), white blood cells (WBC), hemoglobin, hematocrit, red cell distribution width (RDW), albumin, Scr, blood urea nitrogen (BUN), and lactate.

### Outcomes

The primary outcome was the occurrence of AKI within the 7 days of ICU admission; the secondary outcomes were 28- and 90-day mortality from any cause.

### Statistical analysis

We utilized the KNN algorithm for multiple imputations of missing values in the covariates. Variables with a missing rate of more than 20% were eliminated to ensure data reliability.

Only MAP recordings from ICU admission (Hour 0) to Hour 24 were considered. The MAP values during this period were divided into 2-hour intervals, 2-hour MAP missing distribution are shown in Supplementary Figure 1. If multiple measurements were acquired within 2-hour intervals, the analysis was performed on the first measurement obtained. Inaccurate MAP measurements were defined as those with MAP ≤ 0 mmHg or MAP > 200 mmHg, and the proportions are detailed in Supplementary Figure 2. Imputation was not carried out in instances where MAP values were absent at a given time. Instead, missing data were managed by employing a GBTM algorithm that relied on likelihood estimation. This specific methodology is described in the following sections.

We used the GBTM [24], a statistical rather than a theoretical approach, to identify MAP trajectory subtypes based on the preceding 24-h MAP data. Individuals were allocated to their most likely trajectory based on predicted membership probabilities in each identified trajectory group. Model fit was evaluated using metrics such as the Akaike information criterion (AIC), Bayesian information criterion (BIC), sample-adjusted BIC (SABIC), and entropy metrics. We determined the optimal number of clusters based on the principle of lower information criteria, higher log-likelihood, and higher entropy. The grouping of results was evaluated using posterior probabilities, which represented the probability of membership in a particular trajectory group based on estimated model parameters. Acceptable posterior probabilities for each group were at least 70% on average [25]. Clinical interpretability and model simplicity were also taken into consideration. The GBTM method was conducted using the ‘lcmm package’ in R software. Model simplicity and clinical interpretability were also considered.

Demographics, comorbidities, laboratory values, and outcomes were compared among different MAP subphenotypes in patients with heart failure. Additionally, the relationship between MAP subphenotypes and outcomes was also investigated in this patient population. Continuous variables were presented as the mean standard deviation (SD) or median interquartile range (IQR), and the differences in distribution between classes were analyzed using ANOVA or the Kruskal–Wallis test. Categorical variables were presented as frequency and percentage, and the distribution differences among categories were evaluated employing the χ2 test or Fisher’s exact test.

The class with the highest number of participants was selected as the reference group. The Kaplan–Meier (K–M) method was used to plot cumulative incidence curves, and the log-rank test was employed to compare risk differences among the five classes. Cox proportional hazards regression was used to construct four models with increasing covariates. Model 1 was univariate analysis. The laboratory examination was modified in model 2. Model 3 incorporated additional covariables, namely age, gender, and comorbidities, in addition to those already considered in Model 2. Model 4 included BMI and the Charlson Comorbidity Index, alongside the covariables adjusted in Model 3. Hazard ratios (HRs) and 95% confidence intervals (CIs) were calculated to demonstrate the risk of AKI occurrence associated with MAP subphenotypes in heart failure patients. The proportional hazards assumption was satisfied before performing multiple Cox proportional hazards regression.

In the first 7 days of ICU admission, if a patient died before experiencing AKI, they would no longer be at risk of developing AKI. This indicates the presence of competing risk between pre-AKI death and AKI occurrence. In this case, using the Cox proportional hazards model may result in competing risk bias. Therefore, to ensure the reliability of the results, the Fine-Gray subdistribution hazard regression model was employed to build competing risk models for the four models mentioned above.

A doubly robust estimation method was employed in this study to determine the independent association between the five MAP trajectories and the risk of AKI. A propensity score model, including covariates and trajectory categories, was constructed using extreme gradient boosting (XGBoost) and multinomial logistic regression. The estimated propensity scores were then used as weights through inverse probability treatment weighting (IPTW) to allocate study subjects [26]. This method generates a pseudopopulation in which the trajectory categories are unaffected by covariables. XGBoost, an integrated machine learning algorithm that utilizes a gradient boosting framework, is based on decision trees with the ability to handle imbalanced data by adjusting the weights of positive and negative samples [27]. Additionally, each covariate’s contribution to the XGBoost model was assessed. The standardized mean difference (SMD) was calculated to assess disparities between the original and IPTW cohorts, with an SMD greater than 0.1 indicating evidence of imbalance. Cumulative incidence curves were also plotted and a log-rank test was conducted for the IPTW cohort. Univariate and multivariable Cox regressions, adjusting for all covariates, were conducted on the weighted cohort to achieve double robust estimates for different MAP trajectories. This approach allowed inference of the independent association between the five MAP trajectories and the risk of AKI.

Subgroup analysis was conducted to ascertain if the association between different MAP trajectories and AKI occurrence varied across various subphenotypes categorized by age, gender, hypertension, diabetes, renal disease, and Charlson comorbidity index. The Cox regression model was used in subgroup analysis, and the interaction between MAP trajectories and the variable representing the subgroup was evaluated.

Two-tailed P values < 0.05 were regarded as statistically significant. Statistical data analyses were conducted using R v4.2.1 and Stata v15.1.

## Results

### MAP trajectory subphenotypes in heart failure patients

**Figure 1.**
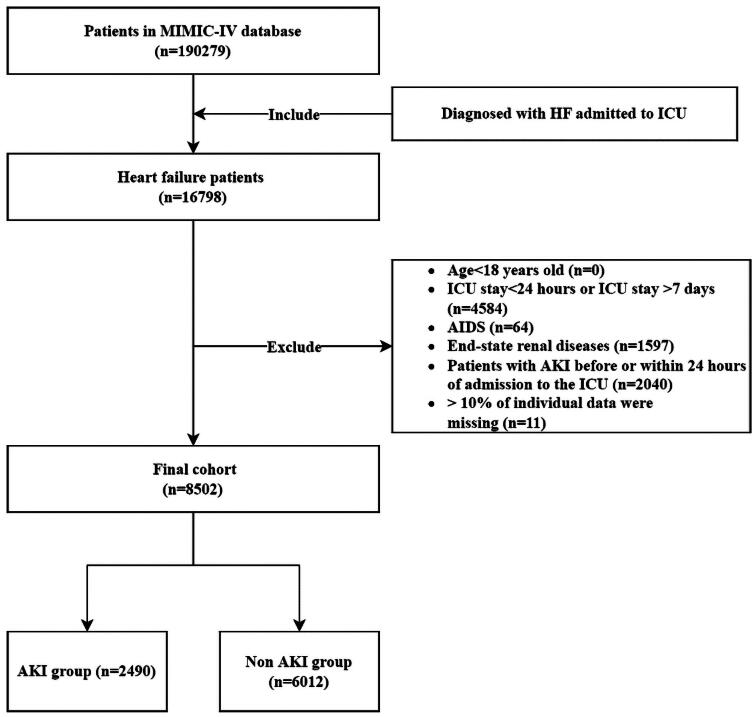
Flow diagram of the patient selection process.

The study cohort consisted of 8,502 patients admitted to the ICU and diagnosed with HF (Figure 1). Among them, 2,490 patients developed AKI within 7 days after ICU admission. The final number of patients included in our cohort fully meets the sample size calculated using the EPV method [3,28,29].

Taking statistical and clinical interpretability into consideration, our model eventually identified five unique trajectory groups with relatively low AIC, BIC, and SABIC values, as well as relatively high log-likelihood ratios (Table 1). The mean posterior probabilities of group membership for the group members were all above 70%, further supporting a great overall fit of the 5-group model (Table 2). The fixed effects of the five-class longitudinal model are detailed in Table 3.

**Table 1.** Comparison of MAP trajectory models.

Number of classes	Log likelihood	AIC	BIC	SABIC	Entropy	%class1	%class2	%class3	%class4	%class5	%class6	%class7	%class8
1	−405088.2	810186.4	810221.6	810205.7	1	100							
2	−392538.1	785096.3	785166.8	785135	0.8870457	71.64197	28.35803						
3	−388769.5	777569	777674.7	777627	0.8339794	40.89626	46.02446	13.07928					
4	−387442	774923.9	775064.9	775001.3	0.8199821	27.40532	47.9299	19.60715	5.057633				
5	−386742.2	773534.4	773710.6	773631.2	0.8008303	26.7937	18.48977	43.14279	6.43378	5.139967			
6	−386158.6	772377.1	772588.5	772493.2	0.7742252	13.13809	40.13173	9.268407	22.90049	11.50318	3.058104		
7	−385710.3	771490.6	771737.3	771626	0.7750081	12.23242	36.93249	26.95836	7.210068	7.033639	6.351447	3.281581	
8	−385415.2	770910.4	771192.3	771065.2	0.7846721	12.50294	36.49729	5.845683	26.26441	3.199247	2.858151	9.668313	3.163961

MAP: Mean Arterial Pressure; AIC: Akaike Information Criterion; BIC: Bayesian Information Criterion; SABIC: Sample-Size Adjusted Bayesian Information Criterion.

**Table 2.** Mean posterior probabilities of MAP subphenotypes.

Class	Probability1	Probability2	Probability3	Probability4	Probability5
Class1	0.8900	<0.0001	0.1041	0.0059	<0.0001
Class2	<0.0001	0.8865	0.0662	0.0282	0.0191
Class3	0.0685	0.0354	0.8486	0.0475	<0.0001
Class4	0.0127	0.0543	0.1403	0.7924	0.0003
Class5	<0.0001	0.0580	<0.0001	0.0011	0.9410

**Table 3.** Fixed effects in the longitudinal five classes model.

Items	Coefficient	Standard error	Wald statistic	*p*-value
Intercept class1	73.09253	0.47523	153.805	<0.001
Intercept class2	93.84631	0.8135	115.362	<0.001
Intercept class3	79.85289	0.52016	153.517	0.308
Intercept class4	119.92614	2.01694	59.459	<0.001
Intercept class5	110.31961	1.04841	105.226	<0.001
poly1 class1	−1.93435	0.14445	−13.391	<0.001
poly1 class2	−1.93023	0.19202	−10.052	<0.001
poly1 class3	−1.18654	0.13795	−8.601	<0.001
poly1 class4	−8.16078	0.48228	−16.921	<0.001
poly1 class5	−2.12109	0.32418	−6.543	<0.001
poly2 class1	0.12623	0.01235	10.221	<0.001
poly2 class2	0.1513	0.01581	9.572	<0.001
poly2 class3	0.07898	0.01097	7.198	<0.001
poly2 class4	0.46582	0.03713	12.546	<0.001
poly2 class5	0.15204	0.02801	5.428	<0.001
Poly3 class1	−0.00248	0.00031	−8.032	<0.001
Poly3 class2	−0.00357	0.00039	−9.087	<0.001
Poly3 class3	−0.00159	0.00027	−5.916	<0.001
Poly3 class4	−0.00879	0.00087	−10.146	<0.001
Poly3 class5	−0.00353	0.0007	−5.014	<0.001

**Figure 2.**
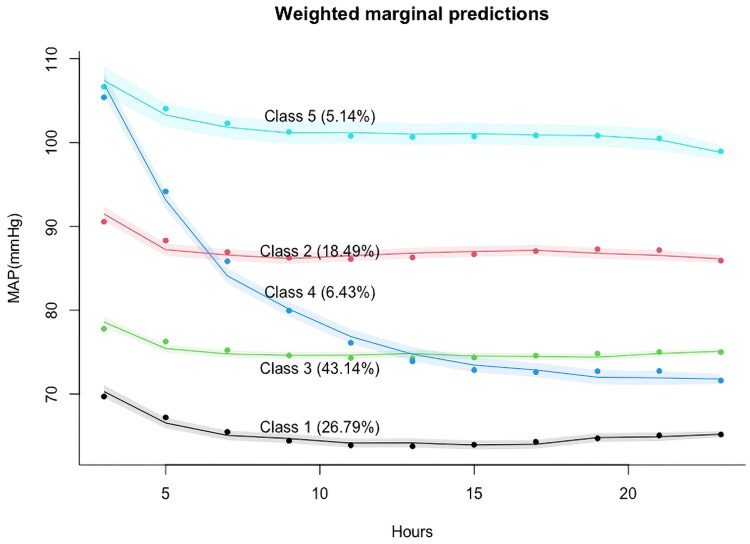
Five classes identified by trajectories of MAP.

The trajectory of MAP subphenotypes is depicted in Figure 2. Each of the five trajectory groups was assigned a descriptive label: Low MAP group (Class 1; 64–69 mmHg), Medium MAP group (Class 2; 85–91 mmHg), Low-medium MAP group (Class 3; 74–78 mmHg), High-to-low MAP group (Class 4; 70–106 mmHg), and High MAP group (Class 5; 99–107 mmHg). Class 1 accounted for 26.79% of the cohort and exhibited consistently low mean arterial pressure. Class 2 and Class 3 demonstrated similar trends at a medium level, characterized by an initial decline followed by a tendency to stabilize. Class 4, accounting for 6.43%, displayed high mean arterial pressure initially, followed by a rapid decline to even lower levels than Class 3. Class 5, representing 5.14%, exhibited persistently high mean arterial pressure with a gradual downward trend.

### Comparisons of patient characteristics between trajectory groups

The baseline data of the five MAP trajectory subtypes are summarized in Table 4. The average age of participants was 73 years, and the majority of patients were males (52.8%). Among the five classes, the patients in Class 1 were the oldest, had the highest prevalence of renal disease, and had the highest Scr and BUN levels. They were followed by Class 4, which had the highest incidence of AKI (34.4%). Class 5 patients were more likely to have concomitant hypertension (85.6%). Additionally, Class 2 patients exhibited a relatively younger age distribution and had the lowest rates of AKI (24.2%), 28-day mortality (11.3%), and 90-day mortality (18.0%).

**Table 4.** Baseline characteristics compared between MAP trajectory subphenotypes.

Characteristic	Overall	Class1	Class2	Class3	Class4	Class5	*P*-value
Age (years)	73.0 [63.0,82.0]	77.0 [68.0,85.0]	70.0 [59.0,80.0]	72.0 [63.0,81.0]	74.0 [64.0,84.0]	66.0 [54.0,77.0]	<0.001
Gender [male, n (%)]	4,488 (52.8)	1,142 (50.1)	848 (53.9)	1,989 (54.2)	258 (47.2)	251 (57.4)	<0.001
BMI	27.8 [23.8,32.9]	27.1 [23.3,31.5]	28.4 [23.9,34.1]	28.0 [24.0,32.8]	27.7 [23.9,33.4]	28.9 [24.1,34.5]	<0.001
Co-morbidities, n (%)							
Hypertension	6,567 (77.2)	1,699 (74.6)	1,248 (79.4)	2,820 (76.9)	426 (77.9)	374 (85.6)	<0.001
Diabetes	3,266 (38.4)	868 (38.1)	617 (39.2)	1,381 (37.6)	229 (41.9)	171 (39.1)	0.366
Renal disease	2,341 (27.5)	744 (32.7)	373 (23.7)	964 (26.3)	164 (30.0)	96 (22.0)	<0.001
Malignant cancer	846 (10.0)	259 (11.4)	158 (10.1)	337 (9.2)	55 (10.1)	37 (8.5)	0.072
Charlson Comorbidity Index	7.0 [6.0,9.0]	7.0 [6.0,9.0]	7.0 [5.0,8.0]	7.0 [5.0,8.0]	7.0 [6.0,9.0]	6.0 [5.0,8.0]	<0.001
WBC (K/µL)	12.1 [8.8,16.6]	12.4 [8.8,17.3]	11.0 [8.4,14.8]	12.6 [9.1,17.3]	11.8 [8.9,16.4]	10.9 [8.6,14.5]	<0.001
Hb (g/dL) 12–16.5	9.9 [8.4,11.4]	9.2 [8.0,10.6]	10.7 [9.1,12.1]	9.7 [8.3,11.2]	10.1 [8.7,11.8]	11.7 [9.7,13.1]	<0.001
HCT (%)	30.1 [25.8,34.8]	28.3 [24.6,32.4]	32.8 [28.2,37.3]	29.8 [25.5,34.1]	30.7 [26.7,35.6]	35.7 [30.2,39.6]	<0.001
RDW (%)	15.3 [14.1,16.9]	15.5 [14.3,17.2]	15.1 [14.1,16.9]	15.2 [14.0,16.9]	15.2 [14.0,16.7]	15.1 [13.8,16.7]	<0.001
ALB (g/dL)	3.3 [3.0,3.6]	3.2 [2.8,3.6]	3.4 [3.0,3.6]	3.4 [3.0,3.7]	3.4 [3.0,3.6]	3.4 [3.1,3.7]	<0.001
Scr (mg/dL) 0.5–1.2	1.2 [0.9,1.6]	1.3 [0.9,1.8]	1.1 [0.8,1.4]	1.1 [0.9,1.5]	1.2 [0.9,1.7]	1.1 [0.8,1.4]	<0.001
BUN (mg/dL)	25.0 [18.0,38.0]	30.0 [20.0,46.0]	23.0 [17.0,34.0]	24.0 [17.0,37.0]	26.0 [18.0,38.0]	22.0 [16.0,30.0]	<0.001
Lac	2.0 [1.5,2.7]	2.0 [1.5,2.7]	2.0 [1.5,2.5]	2.1 [1.6,2.8]	1.9 [1.5,2.6]	1.9 [1.6,2.3]	<0.001
Endpoints (%)							
AKI (%)	2,490 (29.3)	732 (32.1)	381 (24.2)	1,070 (29.2)	188 (34.4)	119 (27.2)	<0.001
28-day mortality	1,219 (14.3)	418 (18.3)	178 (11.3)	483 (13.2)	79 (14.4)	61 (14.0)	<0.001
90-day mortality	1,804 (21.2)	598 (26.3)	283 (18.0)	726 (19.8)	111 (20.3)	86 (19.7)	<0.001

MAP: mean arterial pressure; BMI: body mass index; WBC: white blood cells; Hb: hemoglobin; HCT: hematocrit; RDW: red cell distribution width; ALB: albumin; Scr: serum creatinine; BUN: blood urea nitrogen; Lac: lactate.

### Univariate and multivariate analysis

**Figure 3.**
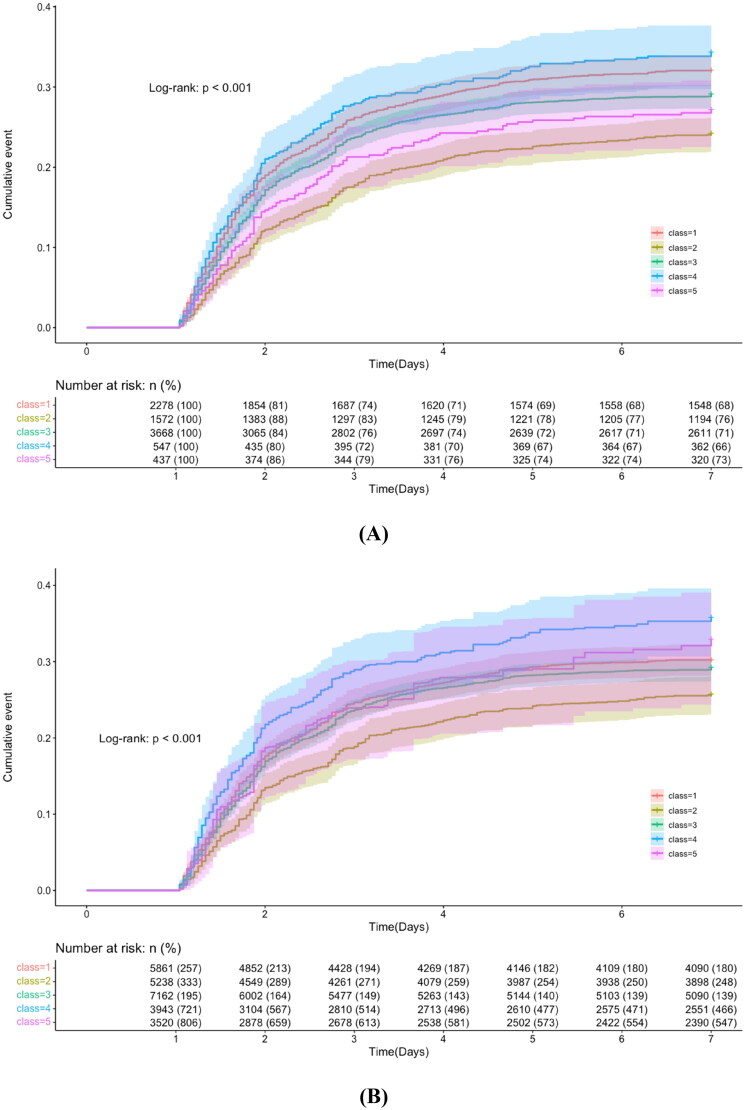
Cumulative incidence curves by Kaplan–Meier method. (A) Before IPTW of AKI, (B) After IPTW of AKI.

Figure 3(A) depicts the cumulative incidence curve generated through the KM method. The statistical analysis using the log-rank test revealed significant differences in the risks of AKI across the five MAP trajectory groups. The risk of Class 4 exhibited a higher magnitude when compared to the remaining classes, while Class 2 had a lower risk than other classes.

Table 5 presents the outcomes of the Cox proportional hazard models. Class 3, the most numerous subphenotype, served as the reference group. In the unadjusted models, belonging to Class 1 and Class 4 was associated with an increased risk of AKI (HR 1.128, 95% CI 1.026–1.239, *p* = 0.012; HR 1.223, 95% CI 1.047–1.427, *p* = 0.011), while belonging to Class 2 was associated with a decreased risk of AKI (HR 0.792, 95% CI 0.705–0.881, *p* < 0.001). Class 5 had a lower hazard ratio (HR) than Class 3, but a statistically significant difference was not observed (*p* > 0.05). After adjusting for potential confounders, the multinomial Cox regression model yielded comparable results, with the exception of Class 1, for which the results were not statistically significant.

**Table 5.** Results of Cox proportional hazard models.

Variables	Model 1		Model 2		Model 3		Model 4	
	HR (95%CI)	P value	HR (95%CI)	P value	HR (95%CI)	P value	HR (95%CI)	P value
Class 1	1.128 (1.026, 1.239)	0.012	1.052 (0.956, 1.157)	0.297	1.049 (0.953, 1.155)	0.328	1.050 (0.954, 1.156)	0.318
Class 2	0.792 (0.705, 0.890)	<0.001	0.875 (0.777, 0.984)	0.026	0.872 (0.774, 0.982)	0.023	0.872 (0.774, 0.982)	0.023
Class 3	Reference		Reference		Reference		Reference	
Class 4	1.223 (1.047, 1.427)	0.011	1.250 (1.067, 1.460)	0.005	1.227 (1.050, 1.433)	0.010	1.221 (1.045, 1.427)	0.012
Class 5	0.913 (0.755, 1.103)	0.342	0.981 (0.806, 1.194)	0.850	0.989 (0.812, 1.204)	0.911	0.994 (0.817, 1.210)	0.954

Adjusted covariates:

Model 1 = Five trajectory subphenotypes.

Model 2 = Model 1 + laboratory examination (Scr, BUN, WBC, Hb, RDW, PLT, Lac, ALB).

Model 3 = Model 2 + age, gender + Co-morbidities (Hypertension, Diabetes, Renal disease, Malignant cancer).

Model 4 = Model 3 + BMI + Charlson Comorbidity Index.

We considered that the competing risk bias brought on by mortality in this research was minimal since only 5% of patients had died before the end of the follow-up period, whereas 29.3% of patients had developed AKI. Consequently, the Fine-Gray proportional subdistribution risk model produced comparable results to the Cox proportional hazard regression in the analysis of competing risk (Table 6).

**Table 6.** Results of fine-gray proportional subdistribution hazard models.

Variables	Model 1		Model 2		Model 3		Model 4	
	HR (95%CI)	P value	HR (95%CI)	P value	HR (95%CI)	P value	HR (95%CI)	P value
Class 1	1.127 (1.026, 1.240)	0.013	1.052 (0.956, 1.157)	0.300	1.049 (0.953, 1.155)	0.330	1.049 (0.953, 1.156)	0.320
Class 2	0.793 (0.706, 0.890)	<0.001	0.875 (0.779, 0.982)	0.023	0.872 (0.777, 0.979)	0.021	0.872 (0.777, 0.979)	0.021
Class 3	Reference		Reference		Reference		Reference	
Class 4	1.222 (1.046, 1.430)	0.011	1.248 (1.068, 1.458)	0.005	1.225 (1.048, 1.432)	0.011	1.220 (1.044, 1.425)	0.012
Class 5	0.913 (0.756,1.100)	0.34	0.982 (0.794, 1.213)	0.860	0.989 (0.804, 1.216)	0.920	0.994 (0.809, 1.222)	0.960

Adjusted covariates:

Model 1 = Five trajectory subphenotypes.

Model 2 = Model 1 + laboratory examination (Scr, BUN, WBC, Hb, RDW, PLT, Lac, ALB).

Model 3 = Model 2 + age, gender + Co-morbidities (Hypertension, Diabetes, Renal disease, Malignant cancer).

Model 4 = Model 3 + BMI + Charlson Comorbidity Index.

### Double robust estimation

**Figure 4.**
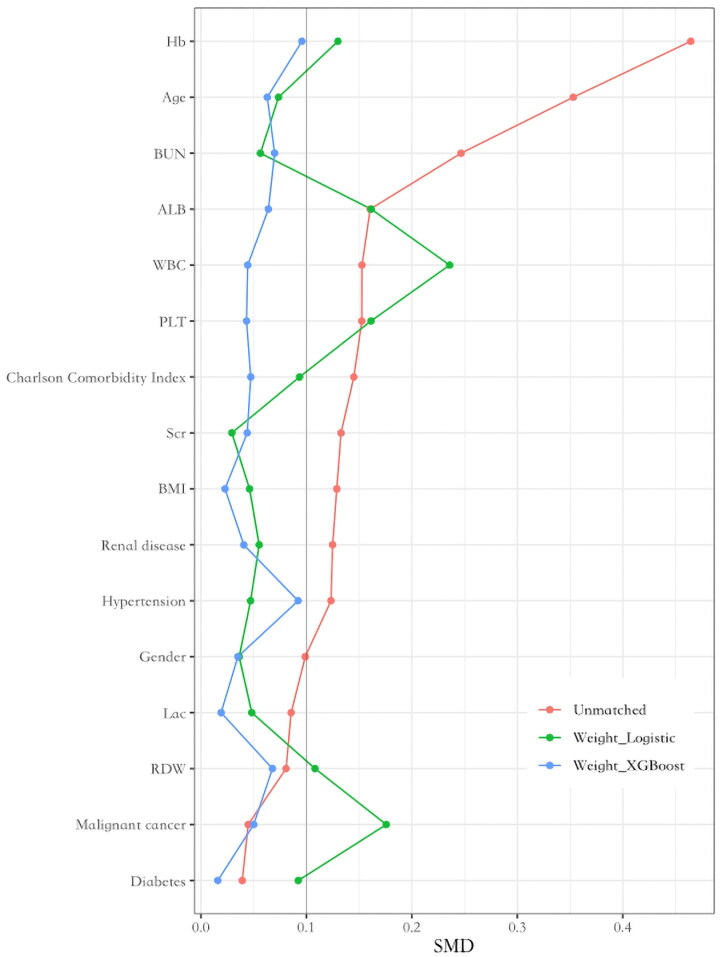
The SMDs of baseline characteristics between three groups.

The covariates across the five MAP trajectory subtypes achieved a well-balanced distribution after IPTW, with XGBoost showing superior performance compared to multinomial logistic regression. After IPTW by XGBoost, the 5 classes were similar on all covariates with standardized mean differences <0.10. Therefore, we chose to conduct a doubly robust analysis using the new cohort derived from IPTW analysis based on XGBoost. Figure 4 presents the standardized mean differences of the original dataset, IPTW dataset using XGBoost, and IPTW dataset using multinomial logistic regression.

Table 7 presents the outcomes of the double robust analysis. In both univariate Cox analysis and multivariable Cox analysis adjusted after IPTW, Class 4 exhibited a significantly increased AKI risk than Class 3 (HR 1.284, 95% CI 1.085–1.521, *p* = 0.003; HR 1.271, 95% CI 1.074–1.505, *p* = 0.005). Conversely, the risks of Class 2 were significantly lower than those of Class 3 (HR 0.846, 95% CI 0.745–0.960, *p* = 0.009; HR 0.879, 95% CI 0.774–0.998, *p* = 0.047). The cumulative incidence curves in the IPTW cohort also exhibited similar patterns to those in the original cohort, and the curves of Class 2 and Class 4 showed an increased distance from the other lines (Figure 3(B)). All of the above indicated consistent and stable results.

**Table 7.** Results of double robust analysis using XGBoost.

Variables	Propensity score IPTW		Doubly robust with all covariates	
	HR (95%CI)	P value	HR (95%CI)	P value
Class 1	1.044 (0.944, 1.155)	0.395	1.030 (0.931, 1.140)	0.554
Class 2	0.846 (0.745, 0.960)	0.009	0.879 (0.774, 0.998)	0.047
Class 3	Reference		Reference	
Class 4	1.284 (1.085, 1.521)	0.003	1.271 (1.074, 1.505)	0.005
Class 5	1.132 (0.860, 1.491)	0.374	1.119 (0.849, 1.476)	0.423

Adjusted covariates:

Model 1(Propensity score IPTW) = Five trajectory subphenotypes.

Model 2 (Doubly robust with all covariates) = Model 1 + laboratory examination (Scr, BUN, WBC, Hb, RDW, PLT, Lac, ALB) + age, gender + Co-morbidities (Hypertension, Diabetes, Renal disease, Malignant cancer) + BMI + Charlson Comorbidity Index.

### Secondary outcome

**Figure 5.**
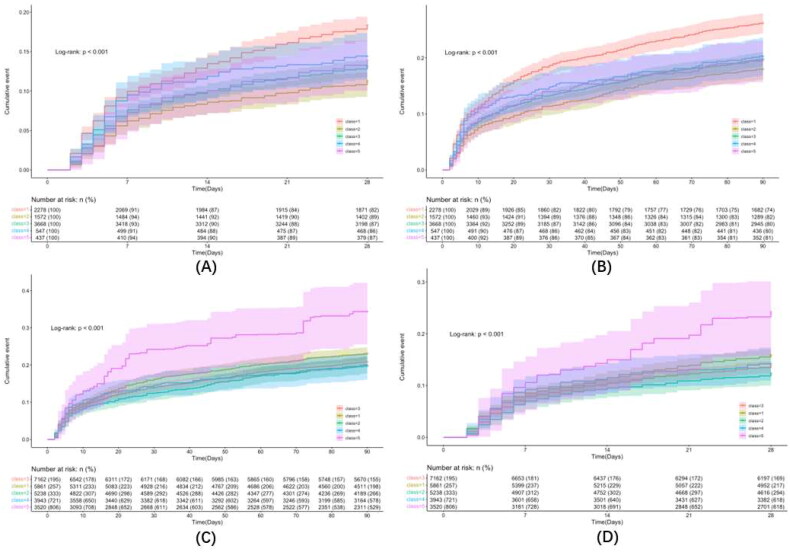
Cumulative incidence curves by Kaplan–Meier method. (A) Before IPTW of 28-day mortality, (B) Before IPTW of 90-day mortality, (C) After IPTW of 28-day mortality, (D) After IPTW of 90-day mortality.

For the secondary outcomes, Cox regression analysis was conducted, and cumulative incidence curves were plotted for both the original and weighted cohorts. Class 1 showed a significantly increased mortality risk at 28 and 90 days than class 3 (HR 1.433, *p* < 0.001; HR 1.384, *p* < 0.001; Table 8), and the results remained robust after IPTW (HR 1.162, *p* = 0.032; HR 1.122, *p* = 0.048; Table 8). Additionally, the mortality rate at 28 and 90 days in Class 5 was higher than that in Class 3 after IPTW, with a larger hazard ratio (HR) compared to Class 1 (HR 1.818, *p* < 0.001; HR 1.769, *p* < 0.001; Table 8). The cumulative incidence curve estimated with the K–M method and the log-rank test result (*p* < 0.001; Figure 5) exhibited a consistent pattern with the findings mentioned above.

**Table 8.** Results of secondary outcomes.

Variables	Class 1		Class 2		Class 3		Class 4		Class 5	
	HR (95%CI)	P value	HR (95%CI)	P value	HR (95%CI)	P value	HR (95%CI)	P value	HR (95%CI)	P value
28-day mortality										
Original cohort	1.433 (1.257, 1.634)	<0.001	0.848 (0.714, 1.007)	0.061	Reference		1.112 (0.876, 1.410)	0.381	1.057 (0.810, 1.380)	0.681
Propensity score IPTW	1.162 (1.012, 1.334)	0.032	0.887 (0.735, 1.069)	0.208	Reference		1.036 (0.799, 1.343)	0.786	1.818 (1.285, 2.570)	<0.001
90-day mortality										
Original cohort	1.384 (1.242, 1.542)	<0.001	0.895 (0.780, 1.027)	0.116	Reference		1.038 (0.850, 1.267)	0.713	0.993 (0.794, 1.242)	0.953
Propensity score IPTW	1.122 (1.001, 1.250)	0.048	0.946 (0.816, 1.096)	0.463	Reference		0.948 (0.761, 1.180)	0.634	1.769 (1.320, 2.370)	<0.001

### Subgroup analysis

The associations between different MAP trajectories and AKI occurrence in prespecified subgroups are reported in Table 9. A consistent correlation was observed between the classification of MAP trajectories and the risk of AKI in heart failure patients with varying characteristics, including age, sex, hypertension, renal disease, and Charlson comorbidity index. The results demonstrate the robustness of our findings. Furthermore, an interaction was observed in the diabetes and nondiabetes subgroups (P interaction = 0.031, not adjusted for multiple testing), suggesting that the relationship between MAP trajectories and AKI may have different effects within this subgroup. Further research and validation are necessary to comprehensively understand the relationship between MAP trajectories and AKI and confirm the clinical significance of the observed differences among the subgroups.

**Table 9.** Results of subgroup analysis.

Subgroups	No. of aki/no. of patients	Class1	Class2	Class3	Class4	Class5	P for interaction
Age							0.423
≥75	1,193/3,979	1.108 (0.973, 1.261)	0.788 (0.657, 0.945)	Reference	1.056 (0.838, 1.331)	0.791 (0.556, 1.126)	
<75	1,297/4,523	1.130 (0.982, 1.299)	0.799 (0.685, 0.931)	Reference	1.390 (1.128, 1.713)	0.982 (0.783, 1.232)	
Gender							0.135
Male	1,320/4,488	1.204 (1.058, 1.371)	0.797 (0.679, 0.935)	Reference	1.453 (1.175, 1.797)	0.865 (0.669, 1.118)	
Female	1,170/4,014	1.050 (0.916, 1.204)	0.786 (0.662, 0.932)	Reference	1.030 (0.821, 1.292)	0.980 (0.740, 1.298)	
Hypertension							0.059
No	507/1,935	0.901 (0.733, 1.106)	0.783 (0.603, 1.018)	Reference	0.890 (0.612, 1.295)	0.880 (0.530, 1.461)	
Yes	1,983/6,567	1.207 (1.085, 1.342)	0.792 (0.695, 0.903)	Reference	1.318 (1.112, 1.564)	0.910 (0.742, 1.118)	
Diabetes							0.031
No	1,441/5,236	1.210 (1.070, 1.368)	0.828 (0.709, 0.966)	Reference	1.135 (0.913, 1.409)	1.109 (0.875, 1.403)	
Yes	1,049/3,266	1.015 (0.876, 1.175)	0.738 (0.617, 0.883)	Reference	1.299 (1.040, 1.622)	0.667 (0.485, 0.918)	
Renal disease							0.235
No	1,567/6,161	1.155 (1.023, 1.303)	0.862 (0.748, 0.993)	Reference	1.189 (0.972, 1.453)	0.918 (0.728, 1.157)	
Yes	923/2,341	0.983 (0.846, 1.142)	0.685 (0.556, 0.845)	Reference	1.213 (0.950, 1.549)	0.986 (0.710, 1.370)	
Charlson comorbidity index							0.341
≥5	2,305/7,643	1.084 (0.984, 1.195)	0.780 (0.689, 0.882)	Reference	1.169 (0.995, 1.373)	0.903 (0.735, 1.110)	
<5	185/859	1.506 (1.001, 2.264)	1.013 (0.697, 1.471)	Reference	1.872 (1.040, 3.369)	1.217 (0.736, 2.010)	

## Discussion

A retrospective study was conducted to investigate heart failure patients admitted to the ICU to assess the relationship between different MAP trajectories and the occurrence of AKI within the initial 7 days. Our findings revealed that compared to Class 3, patients in the Medium MAP group (Class 2) had a significantly reduced incidence of AKI, while those with a swift decline in MAP (Class 4) had a higher risk of AKI.

The results of class 4 were not surprising. Blood pressure variability is associated with the incidence of AKI in critically ill patients [30]. The association between Class 4 and increased AKI risk relates to the concept of autoregulation of blood flow, where arterial blood pressure plays a crucial role in tissue perfusion [10]. When cardiac output remains constant, tissue perfusion remains unaffected until blood pressure falls below a critical threshold. Any additional reduction in arterial blood pressure will impair organ perfusion once this autoregulatory threshold is achieved [31]. The kidney, receiving approximately 25% of the cardiac output, is particularly sensitive to ischemia and hypoxia [32]. We found that approximately one-third of the patients in Class 4 had acute on chronic heart failure (Supplementary Table 1). Unlike chronic or acute heart failure, acute on chronic heart failure leads to a sudden and more severe disruption in hemodynamic stability. The acute increase in fluid overload, worsened ventricular function, and altered neurohormonal activation during an acute exacerbation cause a more pronounced reduction in renal perfusion [33,34]. This inadequate renal perfusion triggers baroreceptors, renin release, and activation of the renin-angiotensin-aldosterone system (RAAS), leading to kidney damage. In addition, lower blood pressure exacerbates renal injury by causing renal hypoxia. This effect is compounded by the combined impact of chronic heart failure’s preexisting renal vulnerability and acute insult, creating an amplified renal injury response. Furthermore, Class 4 and Class 1 patients in our study were characterized by advanced age and the highest incidence of renal disease, both of which have been established as risk factors for AKI in previous studies [23,35]. It should be noted that Class 4 and Class 1 were associated with a higher risk of AKI in the unadjusted model, whereas Class 1 was not statistically significant after adjusting for potential confounders. This may be due to the mediation effect between class 1 and the outcome, which warrants further analysis [33].

A novel finding in our study was the significantly reduced risk of AKI in Class 2 patients compared to Class 3 patients. Although the exact mechanism underlying this observation remains unclear, we hypothesize that a medium MAP level might be appropriate for most heart failure patients, with deviations below or above this level resulting in abnormal renal tissue perfusion and disrupting autoregulation mechanisms and other organ protection processes. The kidney has a higher autoregulatory threshold compared to other organs [36,37], and it is possible that a medium MAP is required for better AKI prevention. Wu et al. observed that maintaining a middle MAP level between 80 and 95 mmHg was associated with a reduced incidence of AKI, while low (65–79 mmHg) and high (96–110 mmHg) MAP levels did not show similar benefits [38], which is consistent with our findings.

However, it is important to note that previous observational studies on critically ill patients identified MAP thresholds for AKI between 65 and 82 mmHg [39,40]. These studies were smaller in scale compared to ours and primarily focused on individuals with septic shock. The population is significant because hypotension is crucial for diagnosing septic shock, possibly leading to AKI [41].

In clinical practice, our findings provide important insights into the hemodynamic management of heart failure patients. When managing heart failure, clinicians should not only focus on the absolute values of blood pressure but also pay closer attention to its dynamic fluctuations and stability. This is especially critical for ICU patients, where significant blood pressure variability may increase the risk of AKI. Therefore, maintaining an optimal mean arterial pressure (MAP) level with precision can help reduce the incidence of AKI and promote the protection of other organs [10]. Additionally, considering the individual differences in heart failure patients, particularly those with comorbidities such as diabetes and hypertension, hemodynamic management may need to be tailored to the individual [42,43]. For certain patients, optimizing pharmacologic therapy (e.g., using vasopressors or diuretics) may be necessary to help maintain the target MAP, preventing inadequate renal perfusion due to low MAP or excessive blood flow and cardiac overload due to high MAP. Finally, our results also suggest that MAP, as a clinical indicator, can not only monitor the hemodynamic status of heart failure patients in real time but also serve as an effective tool for predicting AKI risk by dynamically tracking changes in MAP trajectories, with particular attention to significant variations [44].

Our study also revealed secondary findings of higher 28-day and 90-day mortality rates in heart failure patients with persistently low MAP (Class 1) and persistently high MAP (Class 5).

On the one hand, targeting lower MAP levels may increase the risk of inadequate tissue perfusion and contribute to organ failure progression [39,45]. On the other hand, the higher incidence of concomitant hypertension in Class 5 may be associated with delayed kidney damage in heart failure patients caused by high blood pressure and volume overload-induced hyperfiltration. Moreover, essential organs might suffer damage from elevated blood pressure [46].

This study not only classified MAP trajectories in heart failure patients using GBTM but also analyzed the impact of these trajectories on AKI occurrence using Cox proportional hazards models, competing risk models, and doubly robust estimation methods, ensuring reliable and robust results. Furthermore, future application of our findings can be applied to prospectively predict AKI risk and assist in clinical treatment and decision-making. However, it is important to acknowledge some limitations of our study. First, we did not define AKI events based on urine output according to the KDIGO criteria, which may result in a lower number of patients classified as AKI cases [47]. Second, MAP is influenced by multiple factors, not all of which were accounted for in this study. Third, we observed imbalances and variations in covariates among trajectory classifications, and further research is needed to investigate how these factors affect the dynamic MAP trajectory and its underlying mechanisms. Additionally, an interaction effect was observed in the diabetes and nondiabetes subgroups, necessitating further research and validation to determine the clinical significance of these differences. Finally, we only discussed the effect of MAP trajectory variations on AKI occurrence based on a specific indicator. Future research should consider integrating the common trends of other relevant indicators to predict disease onset [34,48]. Confirmation of our findings in larger, multicenter cohorts of critically ill patients is warranted.

## Conclusion

The trajectory of MAP within 24 h of heart failure patients has a specific effect on the occurrence of AKI. Compared with patients whose MAP was stable at low-medium (74–78 mmHg), patients experiencing a rapid decrease in MAP exhibited a higher risk of AKI, and those with MAP maintained at the medium level (85–91 mmHg) had a significantly reduced risk of AKI. Therefore, to prevent AKI occurrence in heart failure patients, it is necessary to pay close attention to changes in MAP.

## Supplementary Material

Supplementary files.docx

## Data Availability

The datasets generated and/or analyzed during the current study are not publicly available because the data might still be in use for follow-up or related research projects but are available from the corresponding author on reasonable request.

## References

[1] Baman JR, Ahmad FS. Heart failure. JAMA. 2020;324(10):1015. doi: 10.1001/jama.2020.13310. 32749448

[2] Hata N, Yokoyama S, Shinada T, et al. Acute kidney ­injury and outcomes in acute decompensated heart failure: evaluation of the RIFLE criteria in an acutely ill heart failure population. Eur J Heart Fail. 2010;12(1):32–37. doi: 10.1093/eurjhf/hfp169. 20023042

[3] Adams KF, Jr, Fonarow GC, Emerman CL, et al. Characteristics and outcomes of patients hospitalized for heart failure in the United States: rationale, design, and preliminary observations from the first 100,000 cases in the acute decompensated heart failure national registry (ADHERE). Am Heart J. 2005;149(2):209–216. doi: 10.1016/j.ahj.2004.08.005. 15846257

[4] Murugan R, Karajala-Subramanyam V, Lee M, et al. Acute kidney injury in non-severe pneumonia is associated with an increased immune response and lower survival. Kidney Int. 2010;77(6):527–535. doi: 10.1038/ki.2009.502. 20032961 PMC2871010

[5] Sileanu FE, Murugan R, Lucko N, et al. AKI in low-risk versus high-risk patients in intensive care. Clin J Am Soc Nephrol. 2015;10(2):187–196. doi: 10.2215/CJN.03200314. 25424992 PMC4317734

[6] Hsu RK, McCulloch CE, Dudley RA, et al. Temporal changes in incidence of dialysis-requiring AKI. J Am Soc Nephrol. 2013;24(1):37–42. doi: 10.1681/ASN.2012080800. 23222124 PMC3537221

[7] Damman K, Voors AA, Navis G, et al. Current and novel renal biomarkers in heart failure. Heart Fail Rev. 2012;17(2):241–250. doi: 10.1007/s10741-011-9254-2. 21604178 PMC3310988

[8] Soyler C, Tanriover MD, Ascioglu S, et al. Urine neutrophil gelatinase-associated lipocalin levels predict acute kidney injury in acute decompensated heart failure ­patients. Ren Fail. 2015;37(5):772–776. doi: 10.3109/0886022X.2015.1033324. 25869054

[9] Shirakabe A, Hata N, Kobayashi N, et al. Serum heart-type fatty acid-binding protein level can be used to detect acute kidney injury on admission and predict an adverse outcome in patients with acute heart failure. Circ J. 2015;79(1):119–128. doi: 10.1253/circj.CJ-14-0653. 25381804

[10] Sato R, Luthe SK, Nasu M. Blood pressure and acute kidney injury. Crit Care. 2017;21(1):28. doi: 10.1186/s13054-017-1611-7. 28183356 PMC5301320

[11] Khanna AK, Maheshwari K, Mao G, et al. Association between mean arterial pressure and acute kidney injury and a composite of myocardial injury and mortality in postoperative critically ill patients: a retrospective cohort analysis. Crit Care Med. 2019;47(7):910–917. doi: 10.1097/CCM.0000000000003763. 30985388

[12] Lankadeva YR, May CN, Bellomo R, et al. Role of perioperative hypotension in postoperative acute kidney injury: a narrative review. Br J Anaesth. 2022;128(6):931–948. doi: 10.1016/j.bja.2022.03.002. 35465952

[13] Corrêa TD, Vuda M, Takala J, et al. Increasing mean arterial blood pressure in sepsis: effects on fluid balance, vasopressor load and renal function. Crit Care. 2013;17(1):R21. doi: 10.1186/cc12495. 23363690 PMC4056362

[14] Maheshwari K, Nathanson BH, Munson SH, et al. The relationship between ICU hypotension and in-hospital mortality and morbidity in septic patients. Intensive Care Med. 2018;44(6):857–867. doi: 10.1007/s00134-018-5218-5. 29872882 PMC6013508

[15] Sajobi TT, Menon BK, Wang M, et al. Early trajectory of stroke severity predicts long-term functional outcomes in ischemic stroke subjects: results from the ESCAPE trial (endovascular treatment for small core and anterior circulation proximal occlusion with emphasis on minimizing CT to recanalization times). Stroke. 2017;48(1):105–110. doi: 10.1161/STROKEAHA.116.014456. 27924049

[16] Niyonkuru C, Wagner AK, Ozawa H, et al. Group-based trajectory analysis applications for prognostic biomarker model development in severe TBI: a practical example. J Neurotrauma. 2013;30(11):938–945. doi: 10.1089/neu.2012.2578. 23421760

[17] Nagin DS. Group-based trajectory modeling: an overview. Ann Nutr Metab. 2014;65(2-3):205–210. doi: 10.1159/000360229. 25413659

[18] Nagin DS. Group-based modeling of development. Cambridge: Harvard University Press; 2005.

[19] Johnson A, Bulgarelli L, Pollard T, et al. MIMIC-IV (Version 1.0) PhysioNet. 2021. Available from: https://physionet.org/content/mimiciv/1.0/

[20] Wang X, Zhang T, Gao X, et al. Early human albumin administration is associated with reduced mortality in septic shock patients with acute respiratory distress syndrome: a retrospective study from the MIMIC-III database. Front Physiol. 2023;14:1142329. doi: 10.3389/fphys.2023.1142329. 37089426 PMC10119420

[21] Stevens PE, Levin A; Kidney Disease: Improving Global Outcomes Chronic Kidney Disease Guideline Development Work Group Members. Evaluation and management of chronic kidney disease: synopsis of the kidney disease: improving global outcomes 2012 clinical practice guideline. Ann Intern Med. 2013;158(11):825–830. doi: 10.7326/0003-4819-158-11-201306040-00007. 23732715

[22] Zhang L, Xu F, Han D, et al. Influence of the trajectory of the urine output for 24 h on the occurrence of AKI in patients with sepsis in intensive care unit. J Transl Med. 2021;19(1):518. doi: 10.1186/s12967-021-03190-w. 34930308 PMC8686667

[23] Stille K, Kribben A, Herget-Rosenthal S. Incidence, severity, risk factors and outcomes of acute kidney injury in older adults: systematic review and meta-analysis. J Nephrol. 2022;35(9):2237–2250. doi: 10.1007/s40620-022-01381-2. 35932418

[24] Nagin DS, Odgers CL. Group-based trajectory modeling in clinical research. Annu Rev Clin Psychol. 2010;6(1):109–138. doi: 10.1146/annurev.clinpsy.121208.131413. 20192788

[25] Kim SY. Determining the number of latent classes in single- and multi-phase growth mixture models. Struct Equ Modeling. 2014;21(2):263–279. doi: 10.1080/10705511.2014.882690. 24729675 PMC3979564

[26] Bonnot PE, Piessen G, Kepenekian V, et al. Cytoreductive surgery with or without hyperthermic intraperitoneal chemotherapy for gastric cancer with peritoneal metastases (CYTO-CHIP study): a propensity score analysis. J Clin Oncol. 2019;37(23):2028–2040. doi: 10.1200/JCO.18.01688. 31084544

[27] Wang C, Deng C ,Wang S. Imbalance-XGBoost: leveraging weighted and focal losses for binary label-imbalanced classification with XGBoost. Pattern Recognit Lett. 2020;136:190–197.

[28] Peduzzi P, Concato J, Kemper E, et al. A simulation study of the number of events per variable in logistic regression analysis. J Clin Epidemiol. 1996;49(12):1373–1379. doi: 10.1016/s0895-4356(96)00236-3. 8970487

[29] Harrell FE Jr, Lee KL, Mark DB. Multivariable prognostic models: issues in developing models, evaluating assumptions and adequacy, and measuring and reducing errors. Statist Med. 1996;15(4):361–387. doi: 10.1002/(SICI)1097-0258(19960229)15:4<361::AID-SIM168>3.0.CO;2-4. 8668867

[30] Xie Z, Liao X, Yin W, et al. Relationship between short-term blood pressure variability and incidence of acute kidney injury in critically ill patients. Kidney Blood Press Res. 2017;42(6):1238–1246. doi: 10.1159/000485927. 29248933

[31] Demiselle J, Radermacher P, Asfar P. Blood pressure targets in the initial stabilization. In: Pinsky MR, Teboul JL, Vincent JL, editors. Hemodynamic monitoring. Lessons from the ICU. Cham: Springer; 2019. doi: 10.1007/978-3-319-69269-2_29.

[32] Sharfuddin AA, Molitoris BA. Pathophysiology of ischemic acute kidney injury. Nat Rev Nephrol. 2011;7(4):189–200. doi: 10.1038/nrneph.2011.16. 21364518

[33] Schefold JC, Filippatos G, Hasenfuss G, et al. Heart failure and kidney dysfunction: epidemiology, mechanisms and management. Nat Rev Nephrol. 2016;12(10):610–623. doi: 10.1038/nrneph.2016.113. 27573728

[34] De Backer D, Rimachi R, Duranteau J. Hemodynamic management of acute kidney injury. Curr Opin Crit Care. 2024;30(6):542–547. doi: 10.1097/MCC.0000000000001213. 39503203

[35] Küllmar M, Zarbock A, Engelman DT, et al. Prevention of acute kidney injury. Crit Care Clin. 2020;36(4):691–704. doi: 10.1016/j.ccc.2020.07.002. 32892822

[36] Carlström M, Wilcox CS, Arendshorst WJ. Renal autoregulation in health and disease. Physiol Rev. 2015;95(2):405–511. doi: 10.1152/physrev.00042.2012. 25834230 PMC4551215

[37] Bellomo R, Wan L, May C. Vasoactive drugs and acute kidney injury. Crit Care Med. 2008;36(4 Suppl):S179–S86. doi: 10.1097/CCM.0b013e318169167f. 18382191

[38] Wu X, Jiang Z, Ying J, et al. Optimal blood pressure decreases acute kidney injury after gastrointestinal surgery in elderly hypertensive patients: a randomized study: optimal blood pressure reduces acute kidney injury. J Clin Anesth. 2017;43:77–83. doi: 10.1016/j.jclinane.2017.09.004. 29055803

[39] Badin J, Boulain T, Ehrmann S, et al. Relation between mean arterial pressure and renal function in the early phase of shock: a prospective, explorative cohort study. Crit Care. 2011;15(3):R135. doi: 10.1186/cc10253. 21645384 PMC3219004

[40] Poukkanen M, Wilkman E, Vaara ST, et al. Hemodynamic variables and progression of acute kidney injury in critically ill patients with severe sepsis: data from the prospective observational FINNAKI study. Crit Care. 2013;17(6):R295. doi: 10.1186/cc13161. 24330815 PMC4056430

[41] Angus DC, van der Poll T. Severe sepsis and septic shock. N Engl J Med. 2013;369(9):840–851. doi: 10.1056/NEJMra1208623. 23984731

[42] Gallo G, Savoia C. Hypertension and heart failure: from pathophysiology to treatment. Int J Mol Sci. 2024;25(12):6661. doi: 10.3390/ijms25126661. 38928371 PMC11203528

[43] Böhm M, Fitchett D, Ofstad AP, et al. Heart failure and renal outcomes according to baseline and achieved blood pressure in patients with type 2 diabetes: results from EMPA-REG OUTCOME. J Hypertens. 2020;38(9):1829–1840. doi: 10.1097/HJH.0000000000002492. 32618884

[44] Yang WY, Melgarejo JD, Thijs L, et al. Association of office and ambulatory blood pressure with mortality and cardiovascular outcomes. JAMA. 2019;322(5):409–420. doi: 10.1001/jama.2019.9811. 31386134 PMC6822661

[45] Dünser MW, Takala J, Ulmer H, et al. Arterial blood pressure during early sepsis and outcome. Intensive Care Med. 2009;35(7):1225–1233. doi: 10.1007/s00134-009-1427-2. 19189077

[46] James PA, Oparil S, Carter BL, et al. 2014 evidence-based guideline for the management of high blood pressure in adults: report from the panel members appointed to the Eighth Joint National Committee (JNC 8). JAMA. 2014;311(5):507–520. doi: 10.1001/jama.2013.284427. 24352797

[47] Kellum JA, Sileanu FE, Murugan R, et al. Classifying AKI by urine output versus serum creatinine level. J Am Soc Nephrol. 2015;26(9):2231–2238. doi: 10.1681/ASN.2014070724. 25568178 PMC4552117

[48] Nasa P, Wise RD, Smit M, et al. International cross-sectional survey on current and updated definitions of intra-abdominal hypertension and abdominal compartment syndrome. World J Emerg Surg. 2024;19(1):39. doi: 10.1186/s13017-024-00564-5. 39609850 PMC11605967

