# Supplementary material for: Heart failure subphenotypes based on mean arterial pressure trajectory identify patients at increased risk of acute kidney injury
Source: Ren Fail. 2025 Jan 19;47(1):2452205. doi: 10.1080/0886022X.2025.2452205 (PMC11749146; doi:10.1080/0886022X.2025.2452205)
Supplement: Supplementary files.docx [file IRNF_A_2452205_SM6471.docx]

**Supplementary Figure 1.** **Two-hour MAP Missing Distributon.**

**Supplementary Figure 2. Inaccurate MAP measurements Distribution.**

**Supplementary Table 1. Types of Heart Failure in Class 4.**

| **ICD code** | **Freq.** | **Percent** | **Types of Heart Failure** |
| --- | --- | --- | --- |
| 4280 | 43 | 7.86 | Congestive heart failure, unspecified |
| 4281 | 1 | 0.18 | Left heart failure |
| 39891 | 3 | 0.55 | Rheumatic heart failure (congestive) |
| 40291 | 3 | 0.55 | Unspecified hypertensive heart disease with heart failure |
| 42820 | 1 | 0.18 | Systolic heart failure, unspecified |
| 42821 | 29 | 5.3 | Acute systolic heart failure |
| 42822 | 49 | 8.96 | Chronic systolic heart failure |
| 42823 | 50 | 9.14 | Acute on chronic systolic heart failure |
| 42830 | 11 | 2.01 | Diastolic heart failure, unspecified |
| 42831 | 14 | 2.56 | Acute diastolic heart failure |
| 42832 | 53 | 9.69 | Chronic diastolic heart failure |
| 42833 | 60 | 10.97 | Acute on chronic diastolic heart failure |
| 42841 | 2 | 0.37 | Acute combined systolic and diastolic heart failure |
| 42842 | 8 | 1.46 | Chronic combined systolic and diastolic heart failure |
| 42843 | 12 | 2.19 | Acute on chronic combined systolic and diastolic heart failure |
| I110 | 21 | 3.84 | Hypertensive heart disease with heart failure |
| I501 | 1 | 0.18 | Left ventricular failure |
| I5020 | 3 | 0.55 | Unspecified systolic (congestive) heart failure |
| I5021 | 16 | 2.93 | Acute systolic (congestive) heart failure |
| I5022 | 25 | 4.57 | Chronic systolic (congestive) heart failure |
| I5023 | 34 | 6.22 | Acute on chronic systolic (congestive) heart failure |
| I5030 | 5 | 0.91 | Unspecified diastolic (congestive) heart failure |
| I5031 | 14 | 2.56 | Acute diastolic (congestive) heart failure |
| I5032 | 27 | 4.94 | Chronic diastolic (congestive) heart failure |
| I5033 | 43 | 7.86 | Acute on chronic diastolic (congestive) heart failure |
| I5041 | 3 | 0.55 | Acute combined systolic (congestive) and diastolic (congestive) heart failure |
| I5042 | 3 | 0.55 | Chronic combined systolic (congestive) and diastolic (congestive) heart failure |
| I5043 | 2 | 0.37 | Acute on chronic combined systolic (congestive) and diastolic (congestive) heart failure |
| I509 | 11 | 2.01 | Heart failure, unspecified |
